# Exploring the Links Between Social Exclusion and Substance Use, Misuse, and Addiction

**DOI:** 10.3389/fpsyg.2021.674743

**Published:** 2021-06-18

**Authors:** Eric D. Wesselmann, Leandra Parris

**Affiliations:** ^1^Department of Psychology, Illinois State University, Normal, IL, United States; ^2^School of Education, College of William & Mary, Williamsburg, VA, United States

**Keywords:** social exclusion, trauma-informed approaches, ostracism, interpersonal rejection, substance use

## Introduction

Humans have a central need to belong, their daily efforts predominately focused on forging—and maintaining—stable social connections (Baumeister and Leary, 1995; Lieberman, 2013; Gabriel, 2021). The downside of being inherently social is that interpersonal interactions are not always positive. Situations in which someone feels physically or emotionally separate from others, termed *social exclusion* (Riva and Eck, 2016), are common and aversive. Exclusion often evokes immediate pain sensations (Eisenberger, 2012), unpleasant emotions (e.g., anger, sadness, shame) and threatens core psychological needs beyond belonging, such as needs for positive self-esteem, control, and perceived meaningful existence (Williams, 2009). Excluded individuals often experience loneliness and develop social anxiety, fearing, and expecting future exclusion (Cacioppo and Patrick, 2008). *Chronically* excluded individuals also report experiencing higher levels of depressive symptoms, helplessness, alienation, and perceived existential meaninglessness (Riva et al., 2017). Individuals from stigmatized groups (e.g., immigrants, formerly incarcerated persons) are most likely to experience chronic exclusion (Kurzban and Leary, 2001). In some cases, such as mental illness stigma, specific instances of exclusion may exacerbate symptoms, thus leading to a cycle of further exclusion (Reinhard et al., 2020).

## Social Exclusion, Trauma, and Substance (MIS)Use

We have argued elsewhere (Wesselmann and Parris, 2020) that these various adverse outcomes suggest chronic social exclusion should be treated as a form of *trauma*: a psychological experience involving intense physical or emotional harm that inflicts lasting damage on one's physical or mental health (Substance Abuse Mental Health Services Administration [SAMHSA], 2014, p. 7). Exclusion-based trauma may also dovetail with trauma from other experiences (e.g., military combat experience; Wesselmann et al., 2018), compounding the individual's suffering. As such, it is important for trauma-focused researchers and therapists to investigate the specific trauma sources to better understand the psychological processes and possible solutions.

Several studies have established a connection between trauma (broadly defined) and substance (e.g., alcohol, narcotics) use and *misuse*, the latter generally involving excessive, often repeated, use in ways that do not align with medical or general usage, causing physical or psychosocial harm to self or others (Substance Abuse Mental Health Services Administration, 2014; Roberts et al., 2015). Related to social exclusion, other studies have established correlations between both loneliness (Cacioppo and Patrick, 2008; DeWall and Pond, 2011; Dyal and Valente, 2015) and stigma-based exclusion with reported substance use/misuse (e.g., Scheim et al., 2017). Still, these links are only indirect. Establishing such links causally is difficult, if not impossible, given the ethical and practical issues with studying misuse behaviorally. To our knowledge, there is only one experiment that directly examines substance *use* as a dependent outcome after experiencing social exclusion (Bacon and Engerman, 2018). This study found a marginally significant increase in the amount of alcohol consumed by excluded (vs. included) participants in an online interaction.

However, other studies have examined how the use of analgesic substances affects the immediate psychological impact of exclusion. These studies have built on the general assumption that physical and social pain share similar neurological underpinnings (Eisenberger, 2012). First, researchers demonstrated that participants who ingested acetaminophen (compared to a placebo group) showed reduced reactivity to a social exclusion manipulation (DeWall et al., 2010). Subsequent researchers found that other analgesic substances, such as alcohol (Hales et al., 2015), marijuana (Deckman et al., 2014), and psilocybin (a key chemical in hallucinogenic mushrooms; Preller et al., 2016) also dull the immediate sting of social exclusion. Further, one study found that substance use (i.e., alcohol consumption) is most effective for dulling the pain of exclusion in casual users, whereas heavy users may not receive numbing benefits (Buckingham et al., 2016). Relatedly, neurological research has found heightened activity in brain regions associated with exclusion-related pain and reduced ability in regulating this pain for alcohol-dependent participants (Maurage et al., 2012). Other studies demonstrate differential reactions to exclusion among users of opioids, finding that these individuals can have higher adverse reactions to exclusion than non-users (e.g., Kroll et al., 2019; Carlyle et al., 2020).

Collectively, these studies suggest that individuals who use substances to numb the immediate pain of social exclusion may ultimately face diminishing returns; as they build a tolerance for the substance, they may also experience reduced analgesic effects for dealing with social exclusion, perhaps even heightened sensitivity. Thus, although substance use (no matter how moderate) may be a functional way of dealing with exclusion-related pain in the short-term, it could become habit-forming and lead to addiction.

## Discussion

When someone seeks treatment for substance misuse or addiction, a therapist taking a trauma-informed approach should identify to what degree social exclusion contributes to their presenting concerns. Social exclusion may be the *primary* cause (i.e., chronic social exclusion was the main trauma elicitor), or it may be a *secondary* cause; perhaps their initial trauma elicitor was something other than exclusion, and their subsequent substance misuse has led their previous social support network to exclude them. Trauma-informed treatment approaches often focus on social support as a key factor in trauma recovery (Substance Abuse Mental Health Services Administration, 2014). The therapeutic alliance offers an important source of social support for clients throughout treatment, likely increasing their sense of belonging (Baumeister and Leary, 1995). Further, therapists should consider integrating multiple forms of social support when determining treatment modality and access (e.g., group counseling, visitation during hospitalization) to maintain clients' sense of belonging as much as possible (Riva, 2016).

Unfortunately, not all social support is beneficial. People who misuse substances may surround themselves with others who misuse both to receive validation and satisfy their need for belonging (DeWall and Pond, 2011; Mead et al., 2011, Experiment 4). Thus, therapists may want to address the dual nature these enablers can have on clients, because both aspects likely contribute to any resistance to adjusting clients' social networks.

Additionally, therapists could encourage clients to pursue alternative ways of coping with exclusion (Riva, 2016). For example, people can recover from exclusion by engaging in self-affirmation tasks (Knowles et al., 2010; Hales et al., 2016). However, the effectiveness of self-affirmation can depend upon the source of the exclusion. One study (Stock et al., 2018) examined race-based exclusion, finding that Black participants who were excluded by White computer-controlled players in an online game showed increased vulnerability to substance use, but this effect was mitigated when they could affirm their racial identity. A general self-affirmation task did not have the same protective effect, unfortunately. Other research has found that religious/spiritual interventions can be useful in helping people recover from substance addiction, at least partially through fostering a general sense of interpersonal connectedness (e.g., Piedmont, 2004). Germane to social exclusion research, one study found that prayer can help excluded individuals recover, but that it has its strongest effect for people who are most committed to their religion (Hales et al., 2016); this approach is less helpful for less-religious people. We caution that these studies have examined the utility of these various interventions in laboratory settings only; thus, more research needs to assess their effectiveness within the therapeutic context.

The degree to which current trauma-informed programs address social exclusion is unclear. Most programs (e.g., Jaycox et al., 2012) provide *opportunities* for the therapist to address specific trauma-related concerns, but do not explicitly target clients' experiences with social exclusion. Thus, it is incumbent upon the therapist to ensure assessing the specific impact and salience of social exclusion is included in treatment. This can include helping clients repair damage to social relationships resulting from substance misuse, creating new opportunities for substance-free social inclusion outside the therapeutic context, and ensuring alignment with clients' cultural values, identities, and experiences when exploring group counseling as a means of socially inclusive therapy.

## Author Contributions

All authors listed have made a substantial, direct and intellectual contribution to the work, and approved it for publication.

## Conflict of Interest

The authors declare that the research was conducted in the absence of any commercial or financial relationships that could be construed as a potential conflict of interest.
